# Intrapartum Obstetric Violence and Associated Factors Among Women Attending Delivery Care at Public Health Facilities of Gondar City: A Cross‐Sectional Study

**DOI:** 10.1002/hsr2.72961

**Published:** 2026-08-04

**Authors:** Mequanente Dagnaw, Meera Indracanti, Suleyman Mohammed Arage

**Affiliations:** ^1^ Department of Epidemiology and Biostatistics, Institute of Public Health University of Gondar Gondar Ethiopia; ^2^ Department of Medical Biotechnology, Institute of Biotechnology University of Gondar Gondar Ethiopia; ^3^ Department of Medical Biotechnology, School of Allied and Healthcare Sciences Malla Reddy University Hyderabad Telangana India; ^4^ Department of Public Health, College of Medicine and Health Science Werabe University Worabe Ethiopia

**Keywords:** Addis Ababa, intrapartum, obstetric violence, violence

## Abstract

**Introduction:**

Violence against women is a major human rights violation and public health concern with serious physical, mental, sexual, and reproductive consequences. Intrapartum obstetric violence can damage trust in healthcare providers and discourage women from using facility‐based maternity services, sometimes more than geographic or financial barriers. However, evidence on intrapartum obstetric violence in Ethiopia remains limited.

**Objective:**

This study aimed to assess the prevalence of Intrapartum Obstetric violence and its associated factors among women attending delivery care at public hospitals in Addis Ababa, Ethiopia.

**Methods:**

An institution‐based cross‐sectional study was conducted among 316 postpartum women at six public hospitals in Gondar city from June 11 to July 31, 2023. Study participants were selected using systematic random sampling. Data were collected using an interviewer‐administered, pretested questionnaire conducted in a face‐to‐face setting. Interviews were carried out at a consistent postpartum time point prior to discharge from the health facility. Collected data were checked for completeness, entered into EpiData version 3.1, and exported to SPSS version 25 for analysis. Descriptive statistics were presented using narration, tables, and figures. Bivariate and multivariate logistic regression analyses were performed to identify factors associated with intrapartum obstetric violence. Odds ratios with corresponding 95% confidence intervals were used to assess the strength of associations.

**Result:**

The magnitude of intrapartum obstetric violence was 27.3%. Age of mothers (AOR: 1.24, 95% CI (1.23, 13.95), educational level of mothers AOR: 24, 95% CI (0.02, 0.37), number of delivery (AOR: 1.35, 95% CI (1.20, 1.53), mode of delivery AOR: 0.27, 95% CI (0.18, 0.44) and occurrence of complication AOR; 0.16, 95% CI (0.03, 0.90) were factors that had association with the intrapartum obstetric violence.

**Conclusion:**

More than one‐fourth of participants experienced intrapartum obstetric violence. Maternal age, education, parity, mode of delivery, and complications were significantly associated. Hospitals should implement context‐specific interventions targeting these risk factors to reduce obstetric violence.

**Abbreviations**
ANC: antenatal care
AOR: adjusted odds ratio
COR: crude odds ratio
ETB: Ethiopia Birr
IOV: intrapartum obstetric violence
LMD: last month delivery
MOH: Ethiopia Ministry of Health
OV: obstetric violence
SVD: spontaneous vaginal delivery
WHO: World Health Organization

## 1 Introduction

In 2014 the World Health Organization (WHO) defined obstetric violence as kinds of disrespectful and offensive treatments that represented a considerable lack of respect, which included the following: “physical abuse, profound humiliation, and verbal abuse, coercive or unconsented medical procedures (including sterilization), lack of confidentiality, failure to get fully informed consent, refusal to give pain medication, gross violations of privacy, refusal of admission to health facilities, neglecting women during childbirth to suffer life‐threatening, avoidable complications, and detention of women and their newborns in facilities after childbirth due to inability to pay” [1].

Obstetric violence (OV) is a form of gender‐based violence (GBV) that targets pregnant and childbearing women during and beyond the intrapartum period, and this case violates human rights and evidence‐based medicine and hinders the delivery of respectful maternity care (RMC) [2, 3].

Violence against women is largely recognized as a major human rights abuse and a significant public health problem with multiple adverse physical, mental, sexual, and reproductive health effects [4, 5]. A universal human right declares that every childbearing woman in every health system worldwide should practice respect and compassionate care as part of their basic human rights, including respect for women's autonomy, dignity, feelings, and choices [6].

Obstetric violence during facility childbirth includes physical abuse, lack of consent for care, non‐confidential care, undignified care, abandonment, discrimination, and detention in facilities for failure to pay user fees [7]. Disrespectful delivery of care is one type of obstetric violence that threatens human rights to respectful and discrimination‐free care [4]. Disrespectful and abusive care has seven forms, including physical abuse, non‐consented care, non‐dignified care, non‐confidential care, discriminatory care, abandonment, and detention in facilities [8].

The World Health Organization (WHO) defines compassionate and respectful maternal care (CRMC) as the right every woman has to attain the highest standard of health, which similarly includes the right to dignity, compassionate and respectful health care for all childbearing women around the world throughout pregnancy, childbirth, and the postnatal period [1].

Understanding drivers and structural dimensions of mistreatment, including gender and social inequalities, is essential to ensure that interventions adequately account for the broader context [9].

Many women experience disrespectful and abusive treatment during childbirth in facilities worldwide. Such treatment not only violates the rights of women to respectful care but can also threaten their rights to life, health, bodily integrity, and freedom from discrimination [1].

Obstetric violence and mistreatment during childbirth are global problems; however, their worst form is in low‐income countries such as Sub‐Saharan Africa [10, 11, 12, 13, 14]. Violence during pregnancy and delivery has been associated with poor maternal physical health outcomes such as increased sexually transmitted infections (STIs), preterm labor, vaginal bleeding, placental abruption, cesarean delivery, hemorrhage, and infection [15].

The gendered, structural, and institutional nature of obstetric violence during the intrapartum period makes it difficult to recognize due to its widespread normalization and embeddedness in health systems and socio‐cultural norms [16]. The WHO reported that the majority of women are mistreated by their birth attendants. Obstetric violence seems to be widespread throughout the world, with figures ranging from 18.3% [17] to 75.1% [18]. A systematic review conducted in Ethiopia showed that 50% of mothers were disrespected while giving birth in a public institution [19].

Intrapartum violence and abuses create a psychological distance between the women and care providers and then drive women away from formal health care systems in fear of being subjected to such violence, and sometimes are a more prominent hindrance than geographical or financial barriers to maternal health service utilization [17]. Maternal mortality and morbidity are serious public health concerns that have a devastating impact on children, families, and communities worldwide [18, 20, 21].

Sustainable Development Goal 3 aims to reduce maternal mortality below 70 per 100,000 live births. But this ambitious goal cannot be achieved unless the quality of the services is improved [22, 23]. In July 2019, the UN General Assembly issued an important report that indicated the situation of violence against women in reproductive healthcare services, which particularly emphasized attention to birth and OVA. This report stresses mobilizations taking place in many countries against such abusive practices. It proposes adopting an approach based on human rights to the various forms of mistreatment that women have suffered in the obstetric context by insisting on not only violating women's rights to live a violence‐free life but also endangering their right to life, health, their physical integrity, their intimacy, their autonomy, and their right against discrimination [24].

The Ethiopian federal Minister of Health has adopted a new Compassionate and Respectful Care (CRC) approach and started providing in‐service training to all healthcare providers [8]. And develop a 5‐year progress plan, called the Health sector transformation plan, personalized a compassionate, respectful care initiative, offering sector‐wide training for healthcare providers, including addressing the issue of respectful maternity care [25, 26].

The presence of obstetric violence has been associated with determinants such as marital status, age, educational level, socioeconomic level, employment status, race, parity, history of miscarriage, as well as the gender and professional category of the person attending the birth, the type of delivery, and the public or private nature of the childbirth center [27, 28, 29, 30]. Also, different clinical practices, such as giving birth on the delivery table in the lithotomy position, performing an episiotomy without the woman's consent, pressure on the uterine fundus, or carrying out vaginal examinations without the woman's permission, are associated with a higher perception of obstetric violence by women [31]. The WHO intrapartum care guideline recommends respectful maternity care for all women, which is care that maintains “dignity, privacy, and confidentiality, ensures freedom from harm and mistreatment, and enables informed choice and continuous support during labor and childbirth” [32]. Therefore, this study aimed to assess the prevalence and associated factors of OV among intrapartum women in public health hospitals in Addis Ababa, Ethiopia.

## 2 Methods

### 2.1 Study Area and Period

The study was conducted in Gondar city, Ethiopia. The town has a total population of 432,191, and it has six sub‐cities, such as Markie 68229, Jan Tkele 175221, Fasil 68432, Arada 68418, Azezo 110367, Zobele 73798, with a total population and reproductive women [33]. Gondar city has 24 public health facilities (3 hospitals, 8 HCs, and 14 health posts) to offer healthcare services. A total of 3061 HWs with different professional disciplines are working in those health care facilities. The study was conducted from June to July 2023 in Gondar city, Ethiopia.

### 2.2 Study Design

A facility‐based cross‐sectional study was conducted.

### 2.3 Population

#### 2.3.1 Source Population

All women who gave birth in the public hospitals of Gondar city.

#### 2.3.2 Study Population

Selected women who gave birth in public hospitals of Gondar city fulfilled the inclusion criteria during the study period.

### 2.4 Eligibility Criteria

#### 2.4.1 Inclusion Criteria

All mothers who gave birth at selected hospitals during the data collection period were included.

#### 2.4.2 Exclusion Criteria

Those mothers who were critically ill or had a life‐threatening condition were excluded.

### 2.5 Sample Size Determination and Sampling Technique

#### 2.5.1 Sample Size Determination

The sample size was determined using a single population proportion formula by considering a study done on obstetric violence and its associated factors in the Amhara region, as 75.1% [34], and with the assumption of a 95% two‐sided confidence interval and 5% margin of error, was used to determine the sample size. *n* = 287, then by adding a non‐response rate of 10%, the sample size was 316. The final sample size for this study was 316.

#### 2.5.2 Sampling Techniques/Procedure

All public health provider institutions in the Gondar city Administration were included. The study participants were allocated proportionally based on the number of last month's delivery (April 2023) reports in each Hospital. Finally, the study subjects were selected by a systematic random sampling technique. To get the *k* value, dividing the total last month's delivery for the sample size (3338/316) gave a sampling fraction (*K*) of 11. Therefore, the laboring mothers were interviewed every *k* ^th^ of 11 intervals till the total sample size of 316 was reached.

### 2.6 Study Variables

#### 2.6.1 Dependent Variable

Intrapartum obstetric violence (IOV).

#### 2.6.2 Independent Variables

##### 2.6.2.1 The Socio‐Demographic and Economic Status of the Women

Age of the mother, marital status, religion, ethnicity, education attendance, occupation, family monthly income, and residence.

##### 2.6.2.2 Obstetric Characteristics of the Participant

ANC follow‐up, place of ANC, ANC received from, number of ANC visits, gravidity, parity, history of previous institutional delivery, route/type of current delivery, preferred birth position, labor was augmented/induced, time of delivery, number of days stayed at the hospital before delivery.

##### 2.6.2.3 Healthcare Provider‐Related Factors

Number of birth attendants, main birth attendant, sex of the main birth attendant, profession of birth attendants, type of health facility, and provider experiences.

### 2.7 Operational Definitions and Terms

Intrapartum Obstetric violence (IOV): those women who replied “yes” to at least one form of OV from the seven forms, such as physical abuse, non‐consented care, non‐confidential care, non‐dignified care, discriminatory care, neglected care, and detention in the health facility will be labeled as having been subjected to IOV [34].

Verbal abuse: Use of harsh language and threats, and blaming, such as provider use harsh/rude language, judgmental or accusatory comments, threats, or blaming [35].

Physical abuse: The presence of at least one of the following activities by the care provider on the client: beating, threatening with beating, slapping, pinching, restraining or tying down during labor, cutting or suturing of episiotomy cuts or perineal tears without the use of anesthesia, and the use of fundal pressure to fasten the delivery of the baby. A woman who answered “yes” to at least one of the criteria was considered as physically being abused at the time of labor and delivery [34, 35, 36].

Non‐consented care: The presence of at least one of the following: providers not giving women or her relatives proper information about medical procedures, not asking for women's permission to conduct medical procedures such as cesarean sections, episiotomies, hysterectomies, blood transfusions, tubal ligation, augmentation of labor; and coercing women into medical procedures such as a cesarean section. A woman who answered “yes” to at least one of the criteria was considered to have been abused in non‐consented care at the time of labor and delivery [34].

Non‐confidential care: The presence of at least one of the following: giving birth in a public view without privacy barriers such as curtains; and having healthcare providers share sensitive clients' information, such as HIV status, age, marital status, and medical history, in a way that other people who are not involved in their care can hear. A woman who replied “yes” to at least one of the criteria was considered to have been abused in non‐confidential care at the time of labor and delivery [34].

Non‐dignified care: A report by the client about at least one of the following: intentional humiliation, blaming, rough treatment, scolding, shouting at, women not allowed to bring a companion to the labor ward, and ordering to stop crying while they are in labor pain. A woman who replied “yes” to at least one of the criteria was considered to have been abused in non‐dignified care at the time of labor and delivery [34].

Discrimination: Discrimination based on specific client attributes like race, age, HIV/AIDS status, traditional beliefs and preferences, economic status, or educational background. A woman who answered yes to at least one of the criteria was considered to have been abused in discriminatory care at the time of labor and delivery [36].

Neglected care: If there were any of the following practices: leaving the laboring woman alone, women giving birth by themselves at health facilities, failure of caregivers to monitor women in labor, and intervening in life‐threatening conditions. A woman who answered “yes” to at least one of the criteria was considered to have been abused, neglected, or in care at the time of labor and delivery [36].

Detention in facilities: detention of mothers in health facilities because of bills or damage to the property of the health care facility. If a woman answered “yes” to at least two of the criteria, then she would be considered as being abused at the time of labor and delivery [34].

### 2.8 Data Collection Tool and Procedure

A tool to assess the prevalence of Intrapartum Obstetric violence and its associated factors among women attending delivery care at public hospitals in Addis Ababa, Ethiopia was adopted by reviewing different literature [34, 35, 37]. The questionnaire was divided into four parts. The tool entails four parts: part one, socio‐demographic variables; part two, obstetric characteristics‐related factors; part three, Health service system‐related factors; and part four, intrapartum obstetric violence‐related questions.

The data was collected by using face‐to‐face interviewer‐administered structured questionnaires by three diploma midwives and one BSc nurse supervisor who speaks the local language (Amharic). The mother in the postpartum period was informed of all the objectives of the study before giving their consent to participate. The participants who were willing to integrate and sign the informed consent document were then interviewed.

### 2.9 Data Quality Assurance

To ensure the quality of the data, first, the data collection tool was prepared in English, translated into Amharic, and then back to English to check its consistency. Then, training of the data collectors and their supervisors was undertaken for 1 day by the principal investigator on the objectives of the study, the relevance of the study, the methods of data collection, confidentiality of the information, and informed consent. A pre‐test was done by taking 5% of mothers in other non‐selected hospitals before the actual data collection period to see the accuracy of responses, as well as to estimate the time needed, and then the questionnaire was adjusted accordingly. The supervisor checked the data collection process daily. The investigator also takes corrective efforts to boost the validity of the conclusion by reviewing and cross‐checking the questionnaires or data for completeness, accuracy, and consistency daily.

### 2.10 Data Processing and Analysis

The data was checked for completeness and consistency before entering it into the software. Then, it was coded, entered, and verified using EPI‐DATA 3.1 software. Then the entered data was exported to SPSS version 25 for analysis. Descriptive statistics were used to summarize the data, and the results were presented using frequency tables and graphs. Factors associated with the dependent variable were assessed using binary logistic regression, after basic assumptions for binary logistic regression and model fitness were checked before running multiple logistic regression analyses. The variables with *p* values ≤ 0.25 were considered candidate variables for multivariate logistic regression analysis. The degree of association between dependent and independent variables was measured using an adjusted odds ratio with a 95% confidence interval, and a *p* value ≤ 0.05 was considered statistically significant.

## 3 Results

### 3.1 Socio‐Demographic Characteristics

A total of 316 eligible mothers were approached in this study, and 308 mothers agreed to participate, which made the response rate of 97.4%. The mean age of participants was 28.2 SD ± 5.5 years. The majority, 128 (41.6%) of study participants were found between the 25–29 age group, and only 67 (21.8%) of them came from outside of Addis Ababa. Among 308 mothers, only 36 (11.7%) were single. Regarding educational and occupational status, the majority of them, 145 (47.1%), completed secondary and above educational level, and 131 (42.5%) were housewives in occupation. Among study participants, 206 (66.9%) of mothers had a monthly income of less than 5000 Birr per month (Table 1).

**Table 1:** Socio‐demographic and economic characteristics of mothers who gave birth in public health facilities of Gondar City, Ethiopia, 2023.

Variables	Category	Frequency	Percent
Age	< 24 years	75	24.4
	25–29 years	128	41.6
	> = 30 years	105	34.1
Residence	Urban	241	78.2
	Rural	67	21.8
Education level	Illiterate	52	16.9
	Primary education	111	36.0
	Secondary and above	145	47.1
Marital status	Married	272	88.3
	Others^a^	36	11.7
Occupation	Housewife	131	42.5
	Government	82	26.6
	Private employee	68	22.1
	Others^b^	27	8.8
Monthly income	< 5000	206	66.9
	> 5000	102	33.1

^a^ Others (single, widowed, and divorced).

^b^ Others (occupation): merchant, daily laborer.

### 3.2 Obstetric Characteristics of the Study Participants

In the current study, most of the women, 262 (85.1%), had a history of ANC follow‐up, but only 62 (23.6%) of them had more than four ANC follow‐up care. Concerning the mode of delivery, nearly half 148 (48.1%) of mothers delivered by C/S. More than half, 169 (54.9%) of them stayed for more than 24 h at the health facility. Furthermore, 151 (49%) had encountered an incidence of complication either in them or on their infant, but only 29 (9.4%) of them were dissatisfied with the overall care (Table 2).

**Table 2:** Obstetric characteristics of mothers who gave birth in public health facilities of Gondar City, 2023.

Variables	Category	Frequency	Percent
ANC follow‐up in the last birth	Yes	262	85.1
	No	46	14.9
Number of ANC visits	1st visit	75	28.6
	2nd visit	79	30.2
	3rd visit	46	17.6
	4 and above visits	62	23.6
Place of ANC	Health center	192	62.3
	Hospitals	102	33.1
	Others	14	4.5
Ever had a delivery at the current health facility	Yes	118	38.3
	No	190	61.7
Who referred you to the current HF	Self	91	29.5
	From HC	203	65.9
	From the clinic and others	14	4.5
Time of birth of the current delivery	Day	140	45.5
	Night	168	54.5
Mode of delivery	SVD	142	46.1
	C/S	148	48.1
	Assisted	18	5.8
Time stayed at HF	< = 24 h	139	45.1
	> 24 h	169	54.9
Complications in infants and mothers	Yes	151	49.0
	No	157	51.0
Satisfaction with overall care	Strongly satisfied	103	33.4
	Satisfied	151	49.0
	Neutral	25	8.1
	Dissatisfied	21	6.8
	Strongly dissatisfied	8	2.6

### 3.3 Health System Characteristics

In the present study, more than half, 168 (54.5%) of pregnant mothers had ANC follow‐up at health centers, and 170 (55.2%) of them attended by two health professionals, but the majority of them, 160 (51.9%), were attended by doctors. More than half, 168 (54.5%) of health professionals who provided delivery care for mothers were male (Table 3).

**Table 3:** Health care system characteristics of mothers who gave birth in public health facilities of Gondar City, 2023.

Variables	Category	Frequency	Percent
Place of ANC for the last pregnancy	Hospital	117	38.0
	Health centers	168	54.5
	Others	23	7.5
Who assists you Delivery attendant (assistant)	Mid‐wife	122	39.6
	Doctors	160	51.9
	Nurse and others	26	8.4
Number of health professionals involved	One	112	36.4
	Two	170	55.2
	Three	20	6.5
	More than 3	6	1.9
Sex of health professionals who attend	Female	140	45.5
	Male	168	54.5

### 3.4 Prevalence of Intra‐Partum Obstetric Violence and Its Dimension

**Figure 1:**
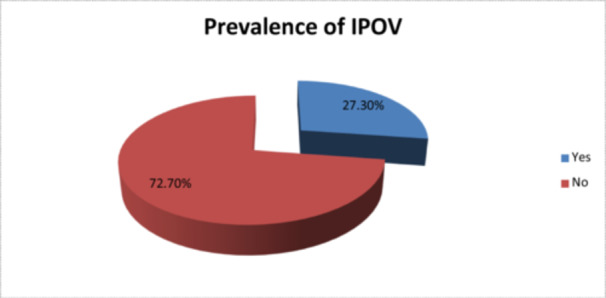
Prevalence of intra‐partum obstetric violence among mothers visiting public health facilities of Gondar City, Ethiopia, 2023.

The findings of this study showed that among the study participants, 84 (27.3%), 95% CI (22.7%, 32.5%) of them reported having encountered intra‐partum obstetric violence (Figure 1).

Concerning the dimension of intra‐partum obstetric violence, 237 (76.9%) of the study participants agreed that the health professionals “Never use physical force or abrasive behavior” while 111 (36%) disagreed that they don't deny food or fluid to women in labor. On the woman's right to information and informed consent related items, 271 (88.0%) agreed on “Allows woman to assume position of choice during birth” related item, while 174 (56.5%) disagreed on “Introduces self to woman and her companion”. Concerning confidentiality and privacy, 263 (85.4%) of mothers agreed that “uses drapes or covering appropriate to protect woman's privacy” while 263 (85.4%) of mothers disagreed. Generally, from IPOV‐related dimension, 245 (79.5%) of mothers agreed on confidentiality and privacy‐related items, while 245 (79.5%) of mothers disagreed on “The woman is detained or confined against her will” (Table 4).

**Table 4:** The proportion of intra‐partum obstetric violence and its components among mothers who gave birth in public hospitals of Addis Ababa, Ethiopia, 2023.

Variables	Yes	No
The woman is protected from physical harm or ill treatment	217 (70.5%)	91 (29.5%)
Never uses physical force or abrasive behavior	237 (76.9%)	71 (23.1%)
Never physically restrains woman	235 (76.3%)	73 (23.7%)
Provides comfort/pain‐relief as necessary	232 (75.3%)	76 (24.7%)
Does not deny food or fluid to women in labor	197 (64.4%)	111 (36.0%)
The woman's right to information and informed consent	197 (64%)	111 (36%)
Introduces himself to the woman and her companion	134 (43.5%)	174 (56.5%)
Encourages the companion to remain with the woman whenever possible	157 (51.0%0	151 (49.0%)
Encourages the woman and her companion to ask questions	224 (72.7%)	84 (27.3%)
Does the provider actively listen to you	244 (79.2%)	64 (20.8%)
Gives periodic updates on the status and progress of labor	229 (74.4%)	79 (25.6%)
Allows the woman to move about during labor	232 (75.3%)	76 (24.7%)
Allows a woman to assume the position of choice during birth	271 (88.0%)	37 (12.0%)
Obtains consent or permission before any procedure	254 (82.5%0	54 (17.5%)
Confidentiality and privacy are protected	245 (79.5%)	63 (20.5%)
Uses curtains or other visual barriers to protect the woman during exams and birth	260 (84.4%)	48 (15.6%)
Uses drapes or a covering to protect the woman's privacy	263 (85.4%)	45 (14.6%)
The woman is treated with dignity and respect	207 (67.2%)	101 (32.8%)
Speaks politely to the woman and the companion	241 (78.2%)	67 (21.8%)
Never makes insults, intimidation, threats, or coerces a woman or her companion	225 (73.1%)	83 (26.9%)
The woman receives equitable care, free of discrimination	131 (42.5%)	177 (57.5%)
Speaks to the woman in a language and at a language‐level that she understands	229 (74.4%)	79 (25.6%)
Does not show disrespect to women based on any specific attribute	157 (51.0%)	151 (49.0%)
The woman is never left without care	156 (50.6%)	152 (49.4%)
Encourages women to call if needed	123 (39.9%)	185 (60.1%)
Never leaves a woman alone or unattended	75 (24.4%)	233 (75.6%)
The woman is detained or confined against her will	82 (26.6%)	226 (73.4%)

### 3.5 Factors Associated With Intra‐Partum Obstetric Violence

In Bivariate binary logistics regression model variables like: age, residence, education level, marital status, occupation, number of delivery, ANC follow up in the last birth, place of ANC, ever had delivery at current health facility, who refer you to current HF, time of birth of the current delivery, mode of delivery, complication in infant and mothers, place of ANC for last pregnancy, number of health professionals and sex of health professionals who attend pass the cut point of binary regression (*p* value ≤ 0.25). These variables were included in the multivariate logistic regression model, and for those variables with *p* < = 0.05 considered as having a significant association with intra‐partum obstetric violence. Predictors of IPOV identified on multivariable regression that were found to be statistically significant at *p* value < 0.05 were: age of the mother AOR = 1.24,95% CI (1.23, 13.95), educational level AOR = 0.24, 95% CI (0.02, 0.37), number of deliveries AOR = (1.35, 95% CI (1.20, 1.53), mode of delivery (0.27, 95% CI (0.18, 0.44)) and complication in infant and mothers AOR = 0.16,95 CI (0.03,0.90) (Table 5).

**Table 5:** Factors associated with intra‐partum obstetric violence among mothers who gave birth in public health facilities of Gondar City, Ethiopia, 2023.

Independent variables	IPOV			COR 95% CI	*p* value	AOR 95% CI	*p* value
		Yes	No				
Age	< 24 years	28	47	1		1	
	25–29 years	37	91	1.47 (0.80, 2.68)	0.215	4.14 (0.99, 5.37)	0.051
	> = 30 years	19	86	2.70 (1.36, 5.34)	0.004	1.24 (1.23, 13.95)	0.005
Residence	Urban	75	166	1		1	
	Rural	9	58	2.91 (1.37, 6.18)	0.005	0.17 (0.02, 1.39)	0.098
Education level	Illiterate	7	45	1		1	
	Primary education	26	85	0.51 (0.21, 1.26)	0.145	1.30 (0.161, 10.56)	0.081
	Secondary and above	51	94	0.29 (0.12, 0.68)	0.005	0.24 (0.02, 0.37)	0.007
Marital status	Married	68	204	1		1	
	Others	16	20	0.42 (0.20, 1.85)	0.016	0.17 (0.03, 1.89)	0.065
Occupation	Housewife	50	81	1		1	
	Government	11	71	3.98 (1.93, 8.24)	0.000	3.08 (0.33, 29.05)	0.327
	Others	23	72	1.93 (1.07, 3.48)	0.028	3.12 (0.54, 27.52)	0.072
number of deliveries	Less than or equal to 3	62	118	1		1	
	Above 3	22	106	2.53 (1.46, 4.40)	0.001	1.35 (1.20, 1.53)	0.0001
ANC follow‐up in the last birth	Yes	78	184	1		1	
	No	6	40	2.83 (1.15, 6.94)	0.023	4.32 (0.76, 24.75)	0.130
Place of ANC	Health center	47	145	1		1	
	Hospitals	30	72	0.79 (0.45, 1.33)	0.360	0.82 (0.08, 8.91)	0.869
	Others	7	7	0.32 (0.11, 0.97)	0.044	0.01 (0.00, 7.97)	0.311
Ever had a delivery at the current health facility?	Yes	27	91	1		1	
	No	57	133	0.69 (0.41, 1.18)	0.174	0.98 (0.14, 6.93)	0.981
Who referred you to the current HF?	Self	31	60	1		1	
	From HC	46	157	1.76 (1.02, 3.04)	0.041	0.95 (0.84, 2.01)	0.71
	From the clinic and others	7	7	0.52 (0.17, 1.61)	0.254	0.38 (0.09, 1.64)	0.195
Time of birth of the current delivery	Day	13	127	1		1	
	Night	71	97	0.14 (0.07, 1.27)	0.043	0.01 (0.0, 1.02)	0.643
Mode of delivery	SVD	24	118	1		1	
	C/S and assisted	60	106	0.36 (0.21, 0.62)	0.000	0.27 (0.18, 0.44)	0.001
Complications in infants and mothers	Yes	30	121	1		1	
	No	54	103	0.47 (0.28, 0.79)	0.005	0.16 (0.03, 0.90)	0.037
Place of ANC for the last pregnancy	Hospital	45	72	1		1	
	Health centers	29	139	2.30 (1.73, 5.18)	0.000	1.17 (0.64, 2.11)	0.063
	Others	10	13	0.81 (0.33, 2.01)	0.653	1.79 (0.11, 2.90)	0.082
Number of health professionals	One	23	89	1		1	
	Two	55	115	0.54 (0.31, 0.95)	0.031	0.65 (0.12, 3.62)	0.622
	Three	6	20	0.86 (0.31, 2.39)	0.775	0.119 (0.03, 4.26)	0.099
Sex of health professionals who attend	Female	25	115	1		1	
	Male	59	109	0.40 (0.24, 0.69)	0.001	1.66 (0.27, 1.03)	0.588

## 4 Discussion

This study tried to assess intrapartum obstetric violence and associated factors among women during childbirth in the study setting. The finding has revealed that the overall level of intra‐partum obstetric violence was 27.3% (95% CI 22.7%, 32.5%). The most reported forms of obstetric violence were detention or confinement against her will, 226 (73.4%), while non‐confidential care, 62 (20.5%), was the least reported. This indicates that almost four women experienced obstetric violence, despite the fact of WHO's statement states that every woman has to be treated in a humanistic and respectful way. The study indicates that the majority of childbearing women experienced a breach of human rights [38]. Obstetric violence intersects with multiple human rights violations (intersectional reproductive health harm) because it encompasses several human rights, including the rights to health, privacy, freedom from discrimination, freedom from violence, and freedom from torture and other ill‐treatment, among others.

The finding of this study was lower than the study done among postnatal women in a Specialized Comprehensive Hospital, Amhara Region, Northwest Ethiopia, which reported an OV of 75.1% [24], and the study done at Gedeo Zone, South Ethiopia, as 79.7% [19]. The observed difference might be the availability of infrastructure and trained human power, and compliance of health professionals with the standard guideline. On the contrary, the finding was higher than the previous community‐based studies conducted in Kenya and Tigray, Ethiopia, in which 20% and 22% of mothers experienced obstetric violence, respectively [38]. This is probably due to the study design used and the time gap between the studies conducted; because as the term obstetric violence is newly recognized as a violation of human rights, mothers may not report it as a violation of their rights rather consider it as standard obstetric care [38] but currently OV is becoming the top concern in different countries regarding empowering child bearing women towards refusing violent care.

Age and maternal educational level were significantly associated with intra‐partum obstetric violence. Compared with women in the early childbearing age group (< 24 years), mothers aged above 30 years had 1.24 times higher odds of experiencing intra‐partum obstetric violence. This finding contrasts with the commonly held assumption that younger mothers are more vulnerable and suggests the need for further interpretation.

One possible explanation may relate to age‐related perceptions or stigma within maternity care settings. Older mothers may be perceived differently by healthcare providers, potentially influencing communication, provider attitudes, or clinical interactions. Such dynamics could contribute to a higher likelihood of negative experiences, including obstetric violence. Future research should explore whether implicit biases or age‐related stigma affect the quality of intrapartum care.

Additionally, maternal age may be interrelated with parity. Women of older age are more likely to have had multiple deliveries, which may influence both provider expectations and clinical decision‐making. To better understand this relationship, we recommend further analysis using multivariate logistic regression that jointly examines maternal age and number of deliveries to assess their independent and combined effects on intra‐partum obstetric violence.

Educational level was another socio‐demographic factor that had a significant association with intrapartum obstetric violence. Being completed secondary school and above were 76% less likely to experience IPOV as compared to the illiterate. This finding is supported by a study done at Gedeo Zone, South Ethiopia [19]. This may be because, as a level of education increases, the probability of mothers being aware of the problem (obstetric violence) would also increase. In addition to this, as the level of education of mothers advances, they become more resistant to accepting violent action during labor and delivery as a normal procedure. So that they become confident enough to state a violent action as violent and thus are more perceptive enough to report all likelihood of being subjected to any form of OV. Moreover, they might develop a good rapport with the provider due to engaging in some level of education and better health‐seeking behaviors that help providers develop a better attitude.

Intrapartum obstetric violence has shown a statistically significant association with obstetric‐related factors such as the number of deliveries, the mode of delivery, and the occurrence of complications.

The odds of having intrapartum violence are 1.35 times more likely among mothers having more than three children than less than three children. This might be due to having more deliveries being related to their age, since those mothers who have more than three children have a decreased ability to push, so that health professionals may use unnecessary words to encourage them to seek help for themselves as well as their children [39].

The odds of having intrapartum violence among women who delivered by using C/S were 73% less likely than SVD. This might be due to the nature of the delivery system. In C/S delivery, there is no need to request a push and other procedures (such as episiotomy) [40].

The odds of having intrapartum violence were 84% less likely in those not having complications in the infant and mothers when compared to those having complications. The possible reason may be that women who develop complications have to stay a longer time in the health facility, increasing their contact time with care providers [41]. These women might suffer secondary pain related to the complication in addition to labor pain. Facing complications can lead to stressed labor and poor maternal health effects as a result of frequent and unnecessary examinations by multiple care providers.

## 5 Conclusion

The findings of this study revealed that the magnitude of obstetric violence during labor and delivery was high, indicating a critical concern for maternal care quality. Detention or confinement of women against their will emerged as a particularly common and serious issue, underscoring the need for immediate corrective measures. Several factors, including maternal age, educational status, parity, mode of delivery, and the occurrence of complications, were significantly associated with experiences of intra‐partum obstetric violence.

These findings highlight the urgent need for institutional and policy‐level interventions. Health institutions should strengthen respectful maternity care (RMC) practices through regular training of healthcare providers, integration of RMC principles into clinical guidelines, and continuous supportive supervision. Establishing clear accountability mechanisms, patient feedback systems, and confidential reporting channels for mistreatment is essential. Additionally, institutional policies must explicitly prohibit detention practices and ensure that women's rights, dignity, and autonomy are upheld.

This study contributes to improving the current situation by providing context‐specific evidence that can guide administrators, policymakers, and professional bodies in designing targeted interventions aimed at reducing obstetric violence and enhancing women's childbirth experiences.

## 6 Study Limitation

This study has some limitations that should be acknowledged. The very short duration of the study period may have restricted the ability to capture variations over time in clinical practices, provider behavior, and women's experiences during labor and delivery. As a result, the findings may reflect conditions specific to the data collection window rather than longer‐term patterns. Future studies conducted over extended periods would provide a more robust understanding of trends and potential seasonal or contextual fluctuations.

## Author Contributions

M.D., M.I., and S.M.A. initiated the idea of research, conceptualizing, developing a proposal, doing analysis, and writing up the manuscript. M.D. contributed to the analysis, reviewing the manuscript and correcting it. Finally, all the authors have approved the manuscript by preparing it for submission. All authors have read and approved the final version of the manuscript.

## Funding

The authors have nothing to report.

## Ethics Statement

Ethical clearance for this study was obtained from the Institutional Review Board (IRB) of the GT Technology College (Reference No. GTTC/26/08/2023). The study was conducted in accordance with the ethical principles outlined in the Declaration of Helsinki (2013 version).

## Consent

Informed consent was not used because the study was retrospective. But by keeping the information that was extracted private and making sure that it could only be utilized for study‐related objectives, the data's secrecy was preserved. The participant's informed consent waiver was authorized by the institutional ethical review board of the GT Technology College in Gondar, Ethiopia.

## Conflicts of Interest

The authors declare no conflicts of interest.

## Transparency Statement

Mequanente Dagnaw affirms that this manuscript is an honest, accurate, and transparent account of the study being reported; that no important aspects of the study have been omitted; and that any discrepancies from the study as planned have been explained.

## Data Availability

The data that support the findings of this study are available from the corresponding author upon reasonable request.
